# Looks Can Be Deceiving: A Case Report on Multicentric Reticulohistiocytosis Successfully Treated with Rituximab

**DOI:** 10.7759/cureus.1220

**Published:** 2017-05-03

**Authors:** Khin Lim, Jason D'Souza, Jonathan B Vasquez, Sujatha Vuyyuru

**Affiliations:** 1 Internal Medicine, Florida Hospital-Orlando; 2 Pathology Department, Orlando VA Medical Center; 3 Rheumatology, Orlando VA Medical Center

**Keywords:** multicentric reticulohistiocytosis, non-langerhan cell histiocytosis, rituximab, papulonodular skin lesions, arthritis mutilans, mrh

## Abstract

Multicentric reticulohistiocytosis (MRH) is an idiopathic multisystemic inflammatory disease characterized by symmetric erosive polyarthritis and typical papulonodular skin lesions. MRH can be associated with autoimmune diseases, malignancy, mycobacterial infections, and hyperlipidemia, and it is important to consider appropriate screening in this population. There is no specific diagnostic laboratory test for MRH. The gold standard for diagnosis is skin or synovial biopsy, which shows characteristic multinucleated non-Langerhans giant cells and ground glass eosinophilic cytoplasm. Although the disease spontaneously remits in approximately 10 years, MRH can rapidly progress to arthritis mutilans in the majority of cases. The diagnosis and treatment are challenging due to its low prevalence and lack of robust guidelines from major rheumatological societies. Corticosteroids and methotrexate are generally first-line treatment options. However, more recently, biologic agents have been increasingly used in refractory cases with some success. Early diagnosis is crucial in preventing disease progression. We report a case of MRH in a patient whose clinical presentation mimicked rheumatoid arthritis and was subsequently treated successfully with rituximab.

## Introduction

Multicentric reticulohistiocytosis (MRH) is a rare multisystemic inflammatory disease characterized by non-Langerhans cell histiocytosis predominantly affecting skin and joints, although it can rarely involve other organ systems. Although its clinical presentation is similar to rheumatoid arthritis (RA) and psoriatic arthritis, the clinical spectrum is more aggressive and the treatment is more challenging. Diagnosis is usually made by the characteristic skin and joint findings, along with the typical histologic appearance of non-Langerhans cell histiocytosis on skin or synovial biopsy. Early diagnosis is crucial to prevent progression to arthritis mutilans. We report a case of an MRH patient whose clinical presentation mimicked rheumatoid arthritis and was successfully treated with rituximab after failure of four different anti-tumor necrosis factor (anti-TNF) agents.

## Case presentation

A 32-year-old Hispanic woman was being managed at her rheumatologist’s office for a provisional diagnosis of rheumatoid arthritis (RA). During this time, she had tried methotrexate and adalimumab with limited success. Given her refractory symptoms, she presented to us seeking a second opinion.

On probing into her symptoms, she stated that her initial symptoms prior to the diagnosis of RA were two hours of morning stiffness and pain, which initially involved her right shoulder only. However, over a period of years, it gradually progressed to involve bilateral wrists, fingers, and knees as well. She also noted skin lesions involving her left elbow and the extensor aspect of bilateral fingers. They were considered to be xanthomas by her previous physicians. Although these lesions were biopsied, the results were inconclusive. Interestingly, she was noted to have evidence of hypercholesterolemia on labs and was started on cholesterol-lowering medication. Her reviews of symptoms were only positive for secondary amenorrhea. She denied any history of inflammatory bowel disease, uveitis, preceding gastrointestinal infection, nail changes, or involvement of her axial joints.

**Figure 1:**
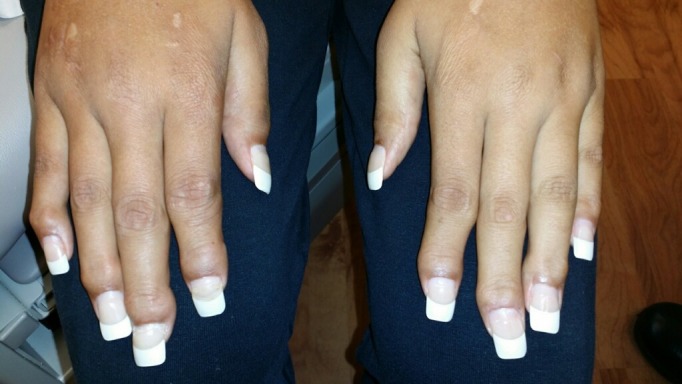
Tenosynovitis of bilateral hand joints

**Figure 2:**
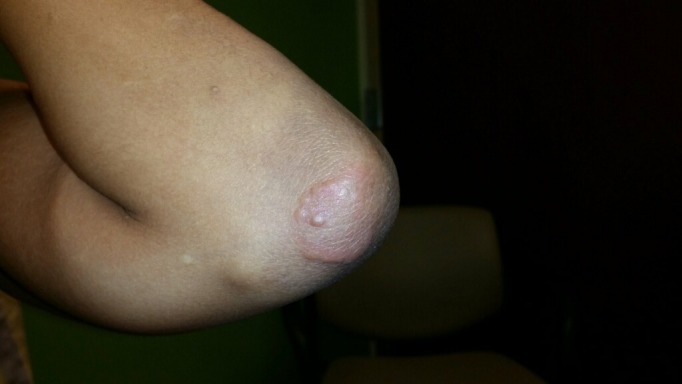
Papulonodular skin lesion on left elbow

On physical examination, there was evidence of synovitis around her right shoulder, right knee, bilateral metacarpophalangeal (MCP) joints, and bilateral proximal and distal interphalangeal (PIP and DIP) joints (Figure 1). Her right shoulder appeared grossly deformed on inspection and palpation, and she had decreased range of motion in all directions. Her left elbow was notable for a flexion contracture and limited range of motion in both flexion and extension. She also had limited range of motion of bilateral MCP, PIP, and DIP joints. She was unable to make a fist in either hand. The examination was also remarkable for the presence of a well-circumscribed papulonodular lesion on the left elbow (Figure 2). Her nails and axial spine appeared to be disease-free. 

**Figure 3:**
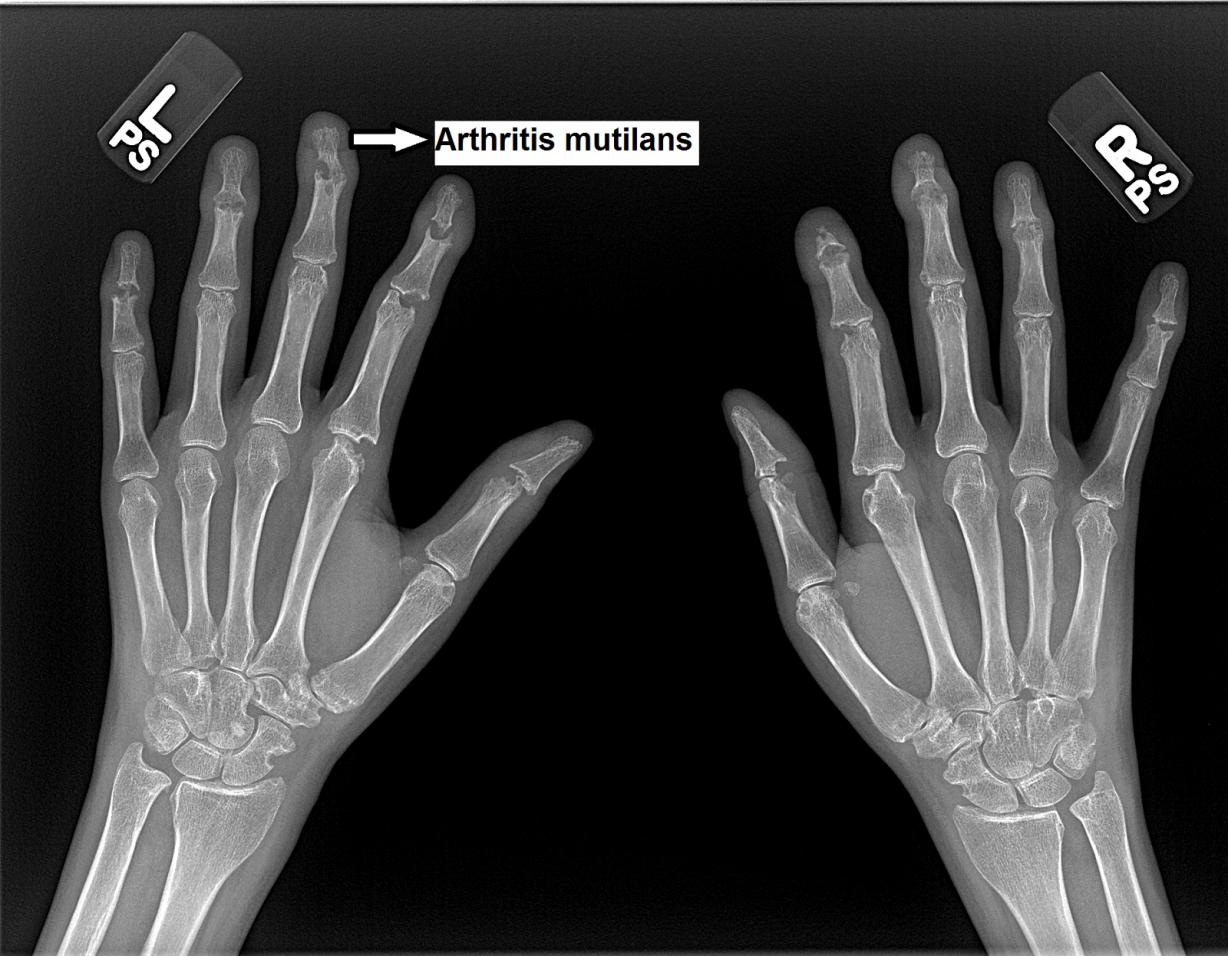
X rays of bilateral hands demonstrates arthritis mutilans mainly affecting bilateral distal interphalangeal joints

Laboratory studies ordered at our office demonstrated a high rheumatoid factor (RF) with 56.4 IU/ml (normal value < 7 IU/ml) and a high erythrocyte sedimentation rate (ESR) of 38 mm/hr. Her C-reactive protein (CRP) and antinuclear antibody (ANA) were normal. Anti-Sjögren’s syndrome related antigen A (anti-SS-A) antibody was elevated with a titer of 8.0 U and anti-Sjögren’s syndrome related antigen B (anti-SS-B) antibody was elevated with a titer of 5.2 U. However, anti-Smith antibodies, anti-double-stranded deoxyribonucleic acid (DNA) antibody (anti-dsDNA), anti-neutrophil cytoplasmic antibody (ANCA), anti-topoisomerase antibody (anti-scl-70), ribonucleoprotein (RNP), anti-Jo1 antibody, and hepatitis panel were negative. With regards to her secondary amenorrhea, she was noted to have a low estradiol level of 11 pg/ml, a high follicle stimulating hormone (FSH) level of 76.6 mIU/ml, and a high luteinizing hormone (LH) level of 62.8 mIU/ml, suggesting premature primary ovarian failure. X-rays of her digits revealed evidence of arthritis mutilans with predominant involvement of her bilateral DIP joints as demonstrated in the image, in addition to the MCP and PIP joints (Figure 3). MRI and x-ray of the right shoulder revealed a widening of the acromioclavicular joint and glenohumeral degenerative changes.

Although her clinical presentation closely fit the diagnosis of RA, not all of her symptoms were explained by it, particularly the involvement of the DIP joints, her skin findings, and the complete lack of response to methotrexate and adalimumab. Nonetheless, she was given the benefit of the doubt, and a trial of multiple other biological agents, such as etanercept, abatacept, and golimumab, was attempted over a period of 12 months. She failed each of these biological agents. She was also noted to have osteopenia and was started on alendronate, but she did not note any change in symptoms with the addition of the alendronate. Owing to the refractory nature of her disease, she was referred to a quaternary center. At the referral center, she was given a trial of rituximab. She demonstrated some clinical improvement of synovitis in her right knee and both hands joints within the first month of rituximab therapy.

**Figure 4:**
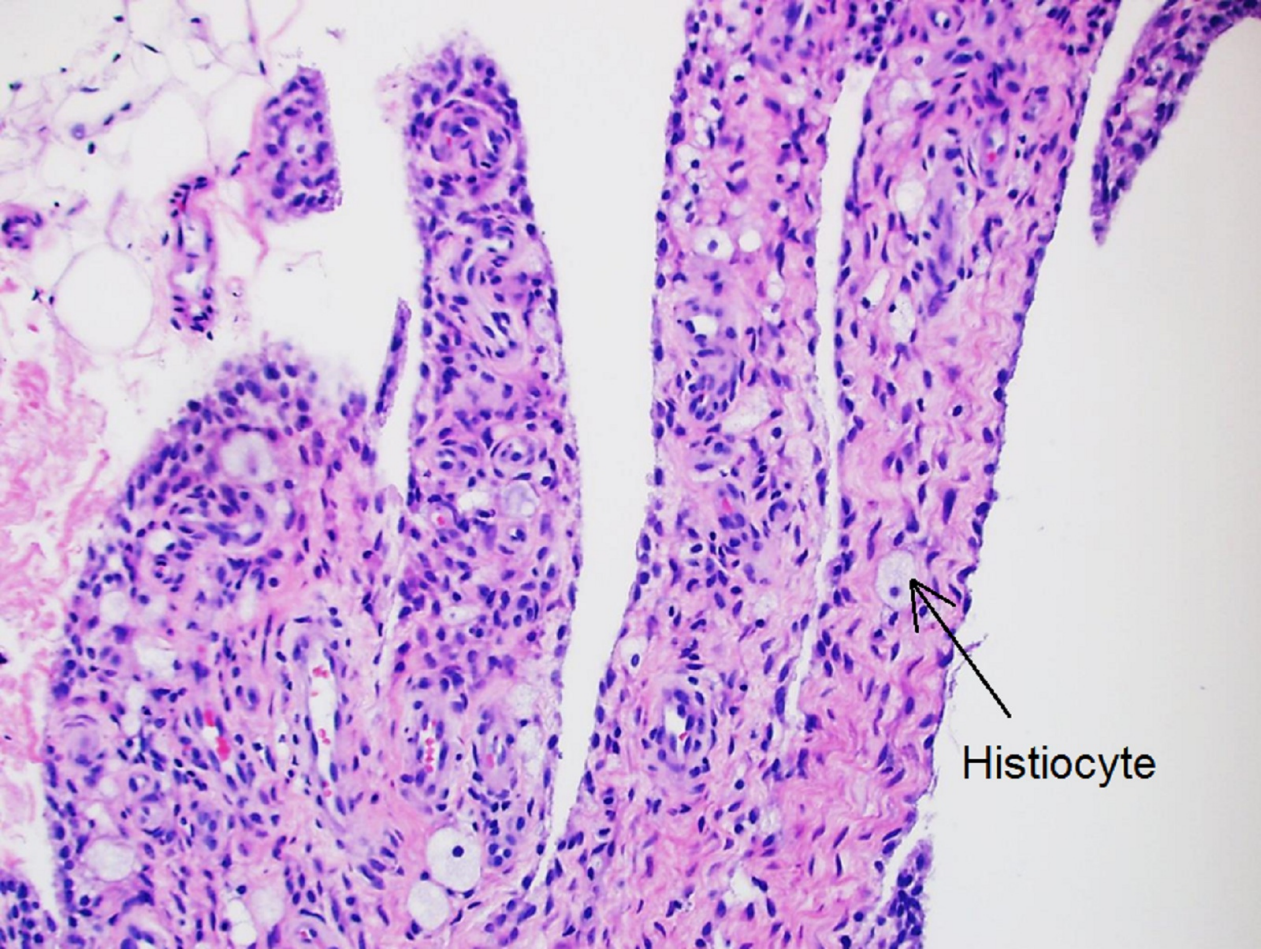
Histologic appearance of the synovium showing histiocytes ​

**Figure 5:**
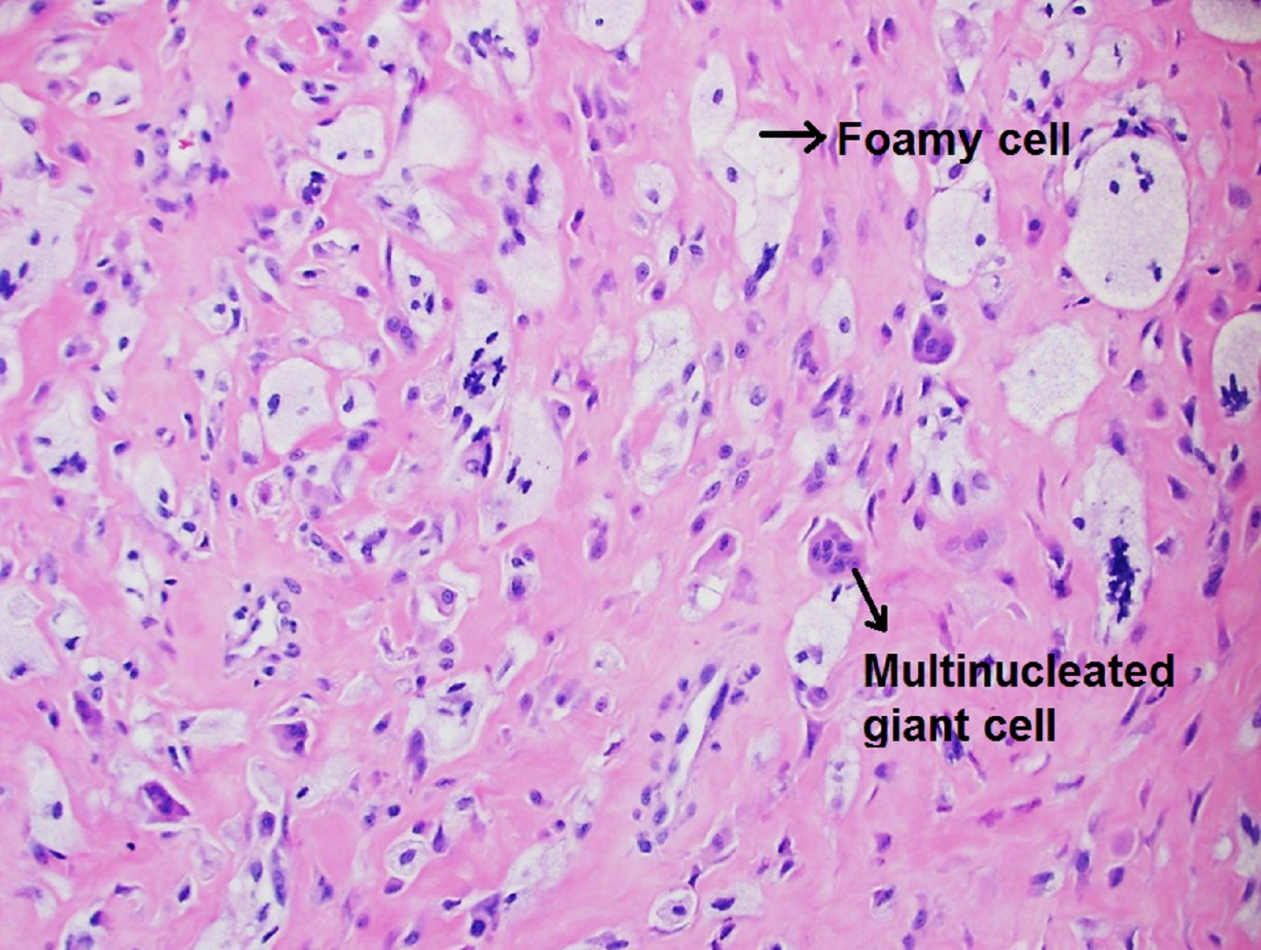
Histologic appearance of the synovium showing foamy cells and multinucleated giant cells

However, due to continuing symptoms in her right shoulder, a synovial biopsy of the right shoulder joint was pursued, which showed evidence of histiocytes and multinucleated giant cells with eosinophilic cytoplasm on histopathology (Figures 4-5). The above findings sealed the diagnosis of multicentric reticulohistiocytosis, which also explained her joints and skin symptoms, the refractory nature to conventional rheumatoid arthritis therapy, hypercholesterolemia in a young lean woman, and could also potentially explain her premature primary ovarian failure. The patient received two infusions of rituximab therapy six months apart. Over a follow-up period of seven months post-rituximab therapy, she noted remarkable improvement in her joint-related symptoms.

## Discussion

Multicentric reticulohistiocytosis (MRH) is a rare form of symmetric erosive polyarthritis associated with morning stiffness and constitutional symptoms that closely resembles rheumatoid arthritis (RA). Originally, it was described as lipoid dermato-arthritis, giant cell reticulohistiocytosis, giant cell histiocytoma, lipid rheumatism, and granulomatous reticulohistiocytosis [1]. It is usually sporadic and can involve various ethnicities, including African-American, Asian, Hispanic, and Native-American populations worldwide. However, most case studies reported a higher incidence in patients of Caucasian ethnicity. This could potentially be attributed to increased awareness in Western countries and availability of sophisticated diagnostic modalities. MRH is more common in women with an approximate gender ratio of 3:1. The mean age at diagnosis is usually in the fourth decade of life [2]. Pathogenesis is idiopathic but is associated with elevated levels of cytokines, including tumor necrosis factor (TNF)-alpha and interleukins (IL), such as IL-1B, IL-6, and IL-8, during the active phase of MRH [3].

MRH is characterized by insidious symmetric inflammatory polyarthritis with both axial and peripheral joint involvement, predominantly in the upper extremities. Hand joints are the most commonly affected joints, particularly the DIP joints, unlike rheumatoid arthritis. The knee joint is the second most commonly affected joint. It can also affect shoulders, hips, ankles, elbows, and feet. The clinical presentation of joint involvement varies from arthralgia and tenosynovitis to erosive arthritis and arthritis mutilans with periarticular osteopenia and deformity in longstanding disease. Cutaneous manifestations are heterogeneous ranging from papulonodular, plaque-like macular erythematous rash, reddish-brown nodules, photosensitive rash, mucosal lesions, xanthelasma to periungual telangiectasia. Lesions can be isolated or scattered and can be accompanied by pruritus. Most commonly affected areas are the face, ears, hands, elbows, shoulders, trunk, and lower extremities. Systemic features, such as weight loss, fatigue, fever, and myalgia, can also be present. Although organ involvement is rare, lungs and cardiac involvement with pleural effusion and pericardial effusion have been reported with sporadic case reports of liver, abdominal, and lymph node involvement as well [4].

Although MRH is isolated and idiopathic, sometimes it has been associated with autoimmune diseases, malignancy, mycobacterial infections, and hyperlipidemia [1]. In our patient, hypercholesterolemia and premature ovarian failure were noted on evaluation. Her low estrogen level, combined with high FSH, LH and normal prolactin levels, are likely due to autoimmune premature ovarian failure associated with MRH. Twenty-five percent of cases are associated with an underlying malignancy and the most common malignancies are hematological, breast, or gastrointestinal tract carcinomas [4]. It is essential to perform malignancy screening and purified protein derivative (PPD) skin test in patients with newly diagnosed MRH. 

Laboratory markers are significant for elevated inflammatory markers and anemia. Rheumatoid factor (RF), antinuclear antibody (ANA), anti-cyclic citrullinated peptide (anti-CCP), anti-double-stranded antibody (anti-ds-DNA), anti-SSA, anti-SSB, and perinuclear anti-neutrophils cytoplasmic antibody (P-ANCA) can also be positive. TNF-alpha levels, proinflammatory cytokines, such as IL-1, IL-6, and IL-8, are also elevated. Diagnosis is usually confirmed by characteristic histiocytes with multinucleated giant cells and ground glass-appearing eosinophilic cytoplasm on the skin and synovial biopsy. The histiocytes contain periodic acid-Schiff (PAS)-positive material with negative S100 and CD1a, indicating the presence of non-Langerhans cells. Treatment response to disease-modifying antirheumatic drugs (DMARDs), cytotoxic, and anti-TNF agents is not consistent. Most of the treatment recommendations come from individual case reports rather than clinical trials due to the rare sporadic nature of the disease. According to a systemic review conducted by Tariq, et al., the most reported effective initial DMARD is methotrexate in combination with prednisone as the first-line therapy [5]. However, our patient did not respond well to these agents and her disease progressed.

Anti-TNF agents are added to individuals who have a suboptimal response. Etanercept, adalimumab, and infliximab have been reported to be effective in some case reports although our patient did not respond well to these agents. Recent case reports also showed satisfactory treatment response with anakinra and tocilizumab [6]. Bisphosphonates can also be added in patients with poor disease control or evidence of osteoporosis or osteopenia. Although the exact mechanism is unclear, it has been assumed that the inhibitory effects on receptor activator of nuclear factor kappa B ligand (RANKL) expressed on histiocytes in the skin and joint of MRH patients may play a role.

Our patient did not initially respond to four biologic agents or bisphosphonates but showed some responses with rituximab. To our knowledge, this is the first case report of treatment of MRH with rituximab. The prognosis of MRH is variable, and in most cases, it remits spontaneously in five to eight years [5].

## Conclusions

MRH is a rare form of symmetric erosive polyarthritis and can easily mimic other autoimmune inflammatory conditions, such as rheumatoid arthritis, psoriatic arthritis, or dermatomyositis. However, MRH has distinct clinical features, such as erosive arthritis involving DIP joints and papulonodular skin findings. Prompt recognition is important for clinicians as untreated cases can progress to severe destructive arthritis and disability.
